# Evidence of amyloid-β cerebral amyloid angiopathy transmission through neurosurgery

**DOI:** 10.1007/s00401-018-1822-2

**Published:** 2018-02-15

**Authors:** Zane Jaunmuktane, Annelies Quaegebeur, Ricardo Taipa, Miguel Viana-Baptista, Raquel Barbosa, Carolin Koriath, Raf Sciot, Simon Mead, Sebastian Brandner

**Affiliations:** 1 Division of Neuropathology, The National Hospital for Neurology and Neurosurgery, University College London Hospitals NHS Foundation Trust, Queen Square, London, WC1N 3BG UK; 2 Department of Molecular Neuroscience, UCL Institute of Neurology, Queen Square, London, WC1N 3BG UK; 3 Portuguese Brain Bank, Neuropathology Unit, Department of Neuroscience, Centro Hospitalar Universitario do Porto, 4099-001 Porto, Portugal; 4 Department of Neurology, Hospital Egas Moniz, Centro Hospitalar de Lisboa Ocidental, 1449-005 Lisbon, Portugal; 5 Department of Neurodegenerative Disease, UCL Institute of Neurology, Queen Square, London, WC1N 3BG UK; 6 Department of Imaging and Pathology, University of Leuven, 3000 Louvain, Belgium; 7 Medical Research Council Prion Unit at UCL, UCL Institute of Prion Diseases, Queen Square, London, WC1N 3BG UK; 8 National Prion Clinic, National Hospital for Neurology and Neurosurgery, UCL Hospitals NHS Foundation Trust, Queen Square, London, WC1N 3BG UK

**Keywords:** Cerebral amyloid angiopathy, CAA, Transmission, Prion diseases, Proteopathic seeding, Amyloid-β, Aβ, Neurosurgery, Decontamination, Intracerebral haemorrhage, Head trauma, Traumatic brain injury, TBI

## Abstract

Amyloid-β (Aβ) is a peptide deposited in the brain parenchyma in Alzheimer’s disease and in cerebral blood vessels, causing cerebral amyloid angiopathy (CAA). Aβ pathology is transmissible experimentally in animals and through medical procedures in humans, such as contaminated growth hormone or dura mater transplantation in the context of iatrogenic prion disease. Here, we present four patients who underwent neurosurgical procedures during childhood or teenage years and presented with intracerebral haemorrhage approximately three decades later, caused by severe CAA. None of these patients carried pathogenic mutations associated with early Aβ pathology development. In addition, we identified in the literature four patients with a history of neurosurgical intervention and subsequent development of CAA. These findings raise the possibility that Aβ pathology may be transmissible, as prion disease is, through neurosurgical procedures.

**Electronic supplementary material:**

The online version of this article (10.1007/s00401-018-1822-2) contains supplementary material, which is available to authorized users.

## Introduction

Prions, the causative agent of prion diseases, are infectious pathogens of humans and animals which are thought to comprise of stable, misfolded and multimeric forms of a normal protein. The paradigm for transmissible human neurodegenerative diseases is the acquired prion disease, such as iatrogenic Creutzfeldt–Jakob disease (iCJD), as a result of prion-contaminated medical procedures, kuru, in the context of ritual cannibalism, or variant CJD due to dietary exposure to bovine spongiform encephalopathy prions [10]. The experimental seeding of amyloid-β (Aβ), the most common misfolded protein in the ageing brain, has been demonstrated in animal models [1, 17, 26].

Recently, we found Aβ in the parenchyma and blood vessel walls in the brains of young patients who had died of iCJD after treatment with cadaveric human pituitary-derived growth hormone (c-hGH) [22]. Subsequently this was confirmed by Ritchie et al. [40], and Cali et al. [7] in c-hGH-treated patients with iCJD and those who did not develop iCJD [40]. The source of the transmitted Aβ was demonstrated by Duyckaerts et al. [13] in batches of growth hormone extracted from pituitary glands which were used for the treatment. Furthermore, significant parenchymal and vascular Aβ pathology has been demonstrated in dura mater grafting-associated iCJD patients [7, 18, 20, 28]. These studies suggest that some aspects of Aβ pathology may be transmissible in certain circumstances. Proteopathic seeding is a useful term to describe these phenomena as it distinguishes these experimental and pathological case observations from the transmission of a fatal neurodegenerative disorder as occurs in prion diseases. Another well-known mode of iatrogenic prion disease transmission is through contaminated neurosurgical instruments [4, 11, 19], which raises the question whether Aβ proteopathic seeds may also be transmitted via this route.

To investigate this possibility, we asked if Aβ pathology occurs in young patients with a past medical history of neurosurgical intervention, and without predisposing genetic risk factors. We searched the pathology archive at our hospital to identify those with pathologically confirmed parenchymal and/or vascular Aβ pathology. In addition, we searched the literature for reported young-onset CAA patients. The search in our archive identified three patients in their thirties and one patient in her fifties, who presented with cerebral amyloid-β angiopathy (Aβ-CAA, in short CAA)-related intracerebral haemorrhages. Four patients were identified from a literature search. Case reports of males in their thirties and forties presenting with spontaneous brain haemorrhages had suggested that the cause of the underlying CAA was male gender and childhood head trauma. Instead, we show here that young-onset CAA is not restricted to male patients with head trauma; rather, we found all cases had a history of neurosurgery during childhood. We demonstrate that similar Aβ pathology is not seen in neurosurgical biopsies of age-matched patients who were treated for various vascular and developmental cortical malformations, and a history of childhood neurosurgery is unusual. We discuss the lack of robust neuropathological evidence of a causal association between a single episode of head trauma and CAA, and raise the possibility that Aβ proteopathic seeds, similar to prions, may be transmissible through surgical instruments.

## Materials and methods

### Case selection and literature search

Informed consent to use the tissue for research was obtained in all cases. Ethical approval for these studies was obtained from the Local Research Ethics Committee of the UCL Institute of Neurology/National Hospital for Neurology and Neurosurgery.

We searched the local pathology archive at our hospital, the National Hospital for Neurology and Neurosurgery (NHNN), from January 2002 to March 2016 for pathologically confirmed parenchymal and/or vascular Aβ pathology on biopsy material. These diagnostic biopsies were obtained during evacuation of intracerebral haematoma or to investigate cognitive decline. Two biopsies were referred from other hospitals. We also searched our pathology archive from January 2007 to December 2017 for pathologically confirmed vascular Aβ pathology on autopsy material. Patients with pathologically confirmed prion disease were excluded from this search as many of these patients have been reported previously [22]. The literature search for young-onset cerebral amyloid angiopathy cases was performed in PubMed (Keywords “young onset” “young”, “early onset” or “early”, “cerebral amyloid angiopathy”, or “CAA”) and subsequent literature was identified from the list of references in these case reports.

As CAA occurs sporadically in individuals above the age of 55 years [27], we studied biopsy samples with Aβ pathology from patients aged 55 and younger.

To investigate whether the history of childhood neurosurgery in our CAA cases was exceptional, we established a control group of 50 consecutive age-matched patients who underwent brain biopsy for vascular or developmental malformations at our hospital (search term “malformation”, date of birth cohort: 1967–1987). In this control group we reviewed the past medical histories and looked for evidence of CAA in brain samples (Supplementary Table 2). Of these 50 cases, 24 were females and 26 were males. The mean age was 34 and median age 32 (range 24–47). These cases were selected to specifically test whether (1) CAA and childhood neurosurgery are associated (our hypothesis), (2) having a brain biopsy in middle age is associated with childhood neurosurgery (alternative hypothesis), or (3) there is no association at all.

### Neuropathological examination

CAA was classified according to the location of the affected vessels (leptomeninges or parenchyma), size (arteries/arterioles and capillaries), extent, and presence of associated vasculopathies [31, 48]. Parenchymal Aβ (diffuse deposits and deposits with central amyloid cores) and tau pathology (neurofibrillary tangles, threads and neuritic plaques, i.e., Aβ plaques with a dense peripheral rim of tau positive neuropil threads) were scored semi-quantitatively. The topographical distribution of parenchymal Aβ in case 4 was assessed according to Thal [45]. The CERAD score was based on neuritic plaque density [34]. Neurofibrillary tangle tau pathology was staged according to Braak and Braak [6].

### Immunohistochemistry

Aβ, hyperphosphorylated tau and macrophages were detected on paraffin tissue sections with antibody 6F3D (DAKO, 1:50), AT8, (Invitrogen MN1020, 1:100) and CD68 (DAKO PG-M1, 1:100), respectively, on a ROCHE Ventana Discovery automated staining platform following the manufacturer’s guidelines, using biotinylated secondary antibodies and a horseradish peroxidase-conjugated streptavidin complex and diaminobenzidine as a chromogen. All immunostainings were carried with appropriate controls.

### Clinical history and genetic testing for risk factors

Past medical history of previous surgical intervention was identified from the clinical notes. The genetic data were sought for mutations in *APP*, *PSEN1*, *PSEN2* genes in Case 1 and Case 2. Next generation sequencing analysis of a panel of 17 genes involved in different forms of dementia [2], including *APP*, *PSEN1*, *PSEN2* genes was carried out on patients reported as Case 3 and Case 4. *APOE* polymorphism was determined in all four patients.

## Results

**Fig. 1:**
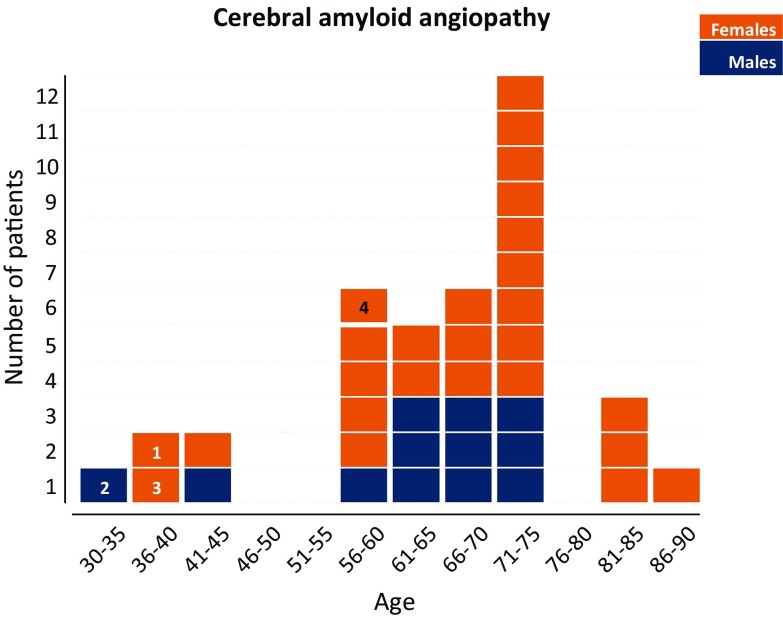
Number of patients with CAA in different age groups over 15-year period from NHNN archive. Histogram showing all patients who underwent surgery, or whose biopsy was reviewed at NHNN during the period from January 2002 to March 2016, and one post-mortem case. In all 37 patients who underwent haematoma evacuation or diagnostic brain biopsy and in one post-mortem case, CAA was histologically confirmed. Cases 1 (#1) and 3 (#3) correspond to the two female patients in the age group 36–40 years, Case 2 (#2) corresponds to the male patient in the age group 30–35, and Case 4 (#4) corresponds to the female patient, aged 57, in whom CAA was diagnosed at autopsy. The two patients in the age group 41–45 correspond to one female patient with a pathogenic mutation in the *PSEN1* gene and one male patient with CAA and parenchymal Aβ pathology in whom further clinical details could not be obtained. Orange, female patients and blue, male patients

In our neuropathology archive, we identified 37 patients operated between 2002 and 2016, aged 31–90 years, with neuropathologically confirmed CAA. Of these, five were younger than 55 years (Fig. 1). Review of the clinical notes showed a pathogenic mutation in the *PSEN1* gene in one of the patients and no clinical information could be obtained from another patient. The remaining three archival cases included two patients referred to us from Portugal and Belgium. All three patients had a neurosurgical intervention during childhood. Whilst it is established that for one of the patients [case 1, (#1)] no dural graft was used during surgery, it is not known if dural grafts were used in the other three patients or blood transfusions had been administered. One patient’s neurosurgery took place in a centre with combined paediatric and adult service, whilst for the other two patients this information could not be obtained. None of these patients had a family history of stroke or dementia, and none had any pathogenic mutation in the *APP*, *PSEN1* and *PSEN2* genes to explain early Aβ pathology. A fourth patient was identified in the autopsy cohort. Review of the pathology notes of all patients who had undergone brain autopsy at our hospital between 2007 and 2017 revealed 45 patients with CAA, one of whom was 57 years old and all others were 63–96 years old. The youngest (57 years old) patient (case 4, (#4) had died from the complications of a large intracerebral haemorrhage with severe widespread CAA confirmed at autopsy. Despite a family history of stroke in this patient, genetic studies did not reveal any pathogenic mutations in the *APP*, *PSEN1* or *PSEN2* genes.

**Fig. 2:**
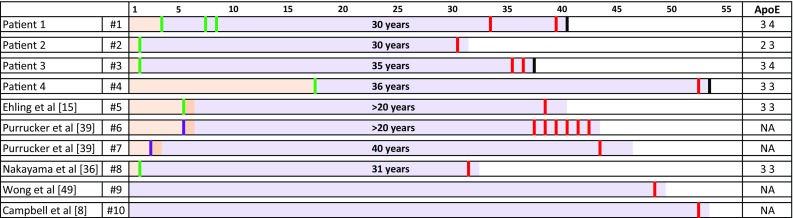
Timeline of the clinical history in patients with CAA: The top row indicates the age in years and the two left columns denote all four patients in our study and reports from the literature, with the patient identifier used in the text. In the timeline, light orange indicates the period prior to neurosurgery; light purple the intervals between neurosurgery and CAA diagnosis or first episode of intracerebral haemorrhage. The green lines on the left indicate the time points of neurosurgical interventions. In patient #5, the time point indicating surgery is an estimate. In patients #6 and #7, the purple lines indicate an estimated time point of head trauma. The red lines on the right indicate the age at which the haemorrhage(s) occurred and the black line indicates the age at death. *ApoE* status for each patient, where available, is indicated in the far right column (*NA* not available). Patient #9 [49] did not have neurosurgery and no clinical information of surgery is available for patient #10 [8]

Below are the descriptions of clinical details and the *APOE* status of these four patients. The case ID numbers (#) correspond to those in Fig. 2 and Supplementary Table 1:

### Case 1 (#1)

**Fig. 3:**
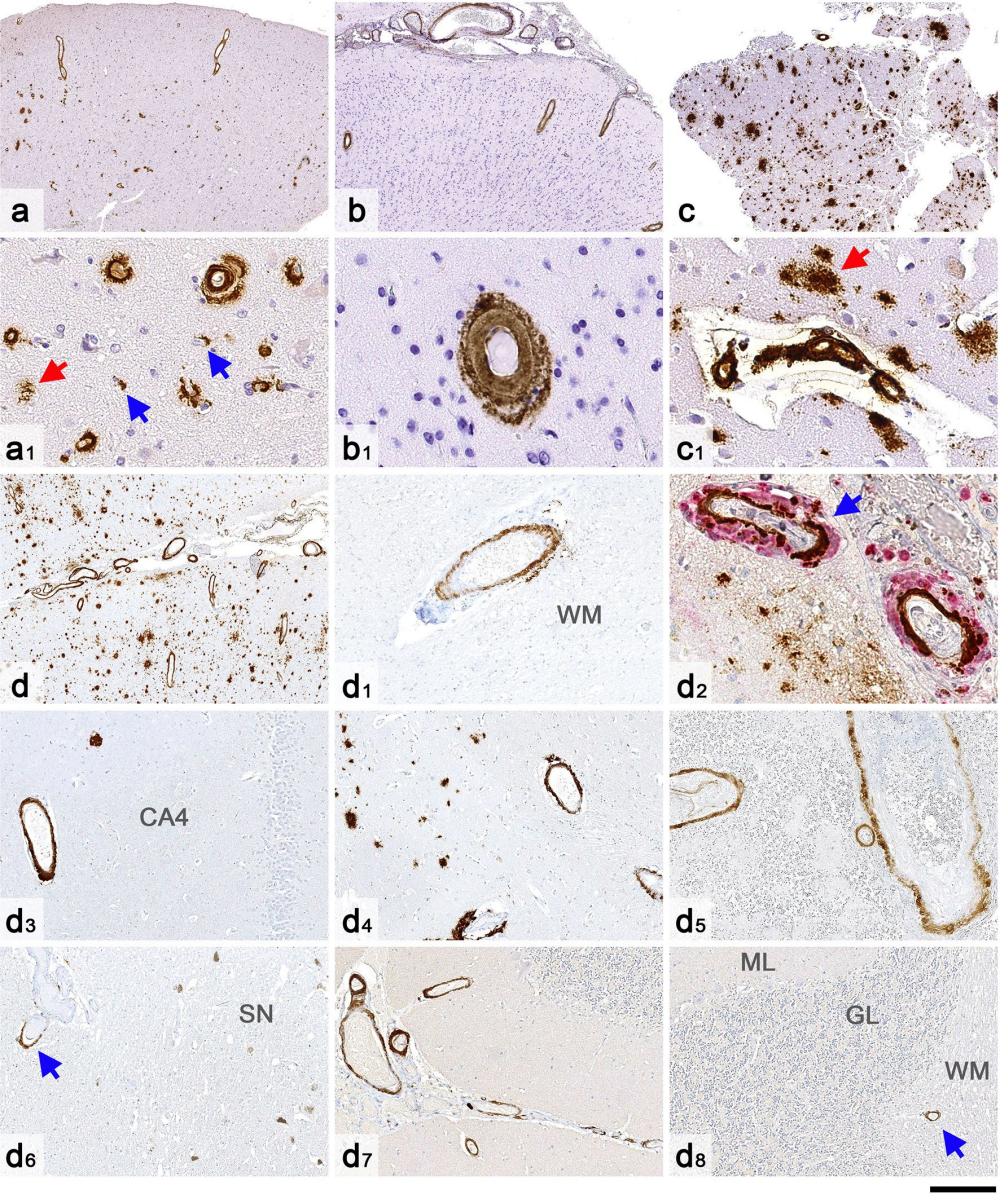
CAA in biopsy and autopsy tissue of the four patients from our cohort. The cortex in case 1 (**a**, **a1**) shows widespread CAA, including capillary involvement (blue arrows in **a1**) and occasional diffuse parenchymal deposits (red arrow in **a1**). In case 2 (**b**, **b1**), there is CAA in leptomeninges and cortex but no diffuse Aβ. In case 3 (**c**, **c1**), there are in addition frequent diffuse parenchymal deposits (red arrow in **c1**). The autopsy case (Patient 4, **d**, **d1**–**d8**) shows widespread CAA in the cerebral leptomeninges and neocortex (**d**) and focally in the cerebral white matter (**d1**, *WM* white matter). In the leptomeninges, there is also focal multinucleated giant cell inflammation (blue arrow in **d2**). Amyloid angiopathy is also present across the medial temporal lobe, including CA4 hippocampal sub-region (**d3**), in the putamen (**d4**), thalamus, including within the haemorrhage (**d5**) and in the midbrain near the substantia nigra (blue arrow in **d6**, *SN* substantia nigra). CAA is also seen in the cerebellar leptomeninges, and to a lesser extent in the cerebellar cortex (**d7**) and focally in the subcortical cerebellar white matter (**d8**; *GL* granule cell layer, *ML* molecular layer). All sections are immunostained for amyloid-β. Section **d2** is double-labelled for amyloid-β (brown) and macrophages (CD68, red). All sections are counterstained with haematoxylin. Scale bar: 450 µm in **a**, **b**, **c**, **d**; 200 µm in **d1**, **d3**, **d4**, **d5**, **d6**, **d7**, **d8** and 50 µm in **a1**, **b1**, **c1** and **d2**

This 39-year-old cognitively intact Portuguese female suffered a severe brain injury at the age of one, requiring multiple cranioplasties at age of three (titanium allograft), seven (platinum allograft) and eight (autograft with iliac bone). Three decades later, at the age of 33, she suffered from her first spontaneous intracerebral haemorrhage. At the age of 39, she presented with a second brain haemorrhage which prompted a diagnostic brain biopsy. In the following 2 months, she suffered further multiple spontaneous bleeds and died soon after. During lifetime, the patient had been extensively investigated, including cerebral angiography on two occasions. Histology of the brain biopsy revealed widespread, severe CAA affecting leptomeningeal and cortical blood vessels, including frequent capillaries in the brain parenchyma, occasional diffuse parenchymal Aβ deposits and rare plaques with central Aβ cores, but no neuritic plaques (CERAD score 0) and no neurofibrillary tangles (Fig. 3). The *APOE* genotype was ε3/ε4.

### Case 2 (#2)

A 31-year-old cognitively intact Belgian male was operated at the age of one on a brain tumour, reportedly a meningioma. At the age of 11, he was involved in an accident requiring intra-abdominal surgery to stop a liver haemorrhage, but no head injury or post-operative neurological complications were reported. The patient presented at the age of 31 with an acute spontaneous parieto-occipital haemorrhage for which he underwent emergency haematoma evacuation and a diagnostic biopsy of surrounding leptomeninges and brain parenchyma. The bleed had developed within and around the resection cavity of the brain tumour. Histology revealed widespread leptomeningeal and parenchymal CAA of moderate severity with no capillary involvement and no parenchymal Aβ deposits (CERAD score 0) (Fig. 3). There was no tau pathology. Further, PiB-PET imaging revealed abnormal signal consistent with deposition of fibrillar Aβ throughout the brain. The *APOE* genotype was ε2/ε3.

### Case 3 (#3)

This 37-year-old British female was born with an Arnold–Chiari malformation, spina bifida and hydrocephalus for which she underwent laminectomy of the cervical spine, repair of a spinal myelomeningocele and ventricular shunting in the first year of life. She presented at the age of 36 with spontaneous left frontal haemorrhage followed by a further bleed in the right frontal lobe several months later, prompting a diagnostic right frontal brain biopsy. In the following months, she had intracerebral haemorrhages at multiple sites, which led to her death at the age of 37. Histology of the brain biopsy showed widespread leptomeningeal and parenchymal CAA of moderate severity, frequent diffuse parenchymal Aβ deposits and several plaques with central amyloid cores. There was no capillary deposition of Aβ (Fig. 3). Tau pathology was restricted to occasional neuropil threads, but no neuritic plaques (CERAD score 0) or neurofibrillary tangles. The *APOE* genotype was ε3/ε4. Next generation sequencing of a panel of 17 genes associated with different forms of dementia revealed a *TREM2* R62H variant, a risk factor for Alzheimer’s disease, but no other alterations.

### Case 4 (#4)

This 57-year-old British female was diagnosed with syringomyelia at age of 17 for which she underwent neurosurgery at age of 20. Subsequently at the age of 40, the patient was also diagnosed with an arteriovenous malformation involving the right insular region, which was initially treated with radiosurgery followed by intravascular coiling 2 years later. The patient presented acutely with a large intracerebral haemorrhage, centred in the left thalamus, but also involving the ventricles, and she died soon after. Post-mortem examination of the brain revealed widespread severe leptomeningeal and parenchymal CAA as the underlying cause of the haemorrhage. There were multiple additional micro-infarcts and micro-haemorrhages across the brain and there was focal leptomeningeal CAA-related inflammation. CAA was also present in cerebral deep grey nuclei (including the origin of the haemorrhage), midbrain parenchyma, and focally in the subcortical cerebral and cerebellar white matter (Fig. 3). There were also widespread parenchymal Aβ deposits corresponding to Thal phase 5 and CERAD score 1, whilst neurofibrillary tangle tau pathology did not exceed Braak and Braak stage II, in keeping with low level Alzheimer’s disease neuropathological change [21, 35]. There was also Lewy pathology restricted to the brainstem, but no evidence of TDP43 proteinopathy. The *APOE* genotype was ε3/ε3. Next generation sequencing of the panel of 17 genes associated with different forms of dementia revealed no pathogenic mutations.

### Case controls

Patients who have a brain biopsy may be more likely to have a history of previous neurosurgery for several reasons, for example, long-term or recurrent conditions, or complications of an old procedure. On review of the brain tissue samples, CAA or parenchymal Aβ pathology was not seen in any of the 50 patients (Supplementary Table 2). In 46 of the 50 age-matched patients, either childhood neurosurgery was specifically stated in the clinical notes not to have happened, or the medical records did not indicate prior neurosurgery. In three of the 50 patients, previous childhood neurosurgery was documented: two patients had intraventricular shunts and one had an anterior communicating artery aneurysm clipped. In one patient, there was a history of severe head trauma, but no details of neurosurgical intervention could be traced. Making a conservative assumption that neurosurgery was done on this patient, four of 50 biopsy patients had evidence of a history of neurosurgery (*P* = 0.0002; 4/50 neurosurgery without CAA vs 4/4 with CAA; Fisher’s exact test).

### Review of the literature

We went on to question whether cases of early onset (age < 55) Aβ CAA were also associated with childhood neurosurgery in the literature. We identified six case reports of CAA in young male adults. Their past medical histories all included a single episode of severe skull bone-penetrating traumatic brain injury (Fig. 2 and Supplementary Table 1). Two of these six individuals definitively had childhood neurosurgery and this was likely, as supported by neuro-imaging, in a further two. One of the six individuals did not have neurosurgery in childhood and details of past medical history were not provided for another one. The age of the patients described below is as stated in the publications, or was calculated from the data provided and in these instances may be approximate.

### Literature case 1 (#5) [15]

This 40-year-old man had a past medical history of traumatic brain injury during childhood, requiring craniotomy. At the age of 38, he developed a left posterior temporal haemorrhage with intraventricular extension. Further haemorrhages occurred in the same year and 2 years later, at the age of 40, requiring haematoma evacuation. Histology confirmed CAA and genetic analysis of *PSEN1*, *PSEN2* and *APP* was negative. *APOE* genotype was ε3/ε3.

### Literature cases 2 (#6) and 3 (#7) [39]

Patient “A” (#6), a 42-year-old male had a history of traumatic brain injury during childhood, with radiological evidence of a residual bone defect suggesting prior surgery. At the age of 37, he presented with the first episode of intracerebral haemorrhage with nine further episodes over the following 6 years. A brain biopsy confirmed CAA. Gene sequencing of *APP* showed no pathogenic mutations.

Patient “B” (#7), a 46-year-old male, suffered from a penetrating head injury at the age of 2 years and presented with a right parietal lobar haemorrhage 40 years later, at the age of 42. Histology confirmed CAA. A further, left temporal haemorrhage with ventricular extension occurred at the age of 46.

### Literature case 4 (#8) [36]

A male, aged 32, had a history of head trauma at the age of one requiring neurosurgical repair. He presented with two episodes of intracerebral haemorrhage, aged 32. Histological and ultrastructural examination of a small tissue sample confirmed CAA and his *APOE* genotype was ε3/ε3.

## Discussion

We present here four young adults of both genders, who presented with CAA-related cerebral haemorrhages. All had undergone neurosurgical procedures several decades earlier, for trauma, correction of a congenital malformation, resection of a brain tumour or syringomyelia. In addition, four young adults with CAA and a history of head trauma were identified in the literature: two of these patients had a documented neurosurgical repair [15, 36] and in two there was circumstantial evidence of neurosurgical intervention [39]. None of the patients from our centre had any known mutations predisposing them to early Aβ pathology such as mutations in the *APP*, *PSEN1* and *PSEN2* genes [9]. Although three out of four patients from our centre were heterozygous for either ε2 or ε4 alleles of *APOE*—known CAA susceptibility alleles [3, 9], widespread CAA and presentation with intracerebral haemorrhages at such a young age would still be highly unusual even for carriers of *APOE* risk alleles. In a study by Pletnikova et al. [37], none of the 154 patients who had died aged between 30 and 50, showed significant CAA: in the stratum of 40–49 years one patient homozygous for *APOE* ε4 had moderate CAA and four patients homo- or heterozygous for *APOE* ε4 had minimal CAA only.

The *TREM2* R62H variant, found in one of our patients, is associated with increased late onset Alzheimer’s disease (AD) risk [23], whilst only the *TREM2* R47H variant is associated with early onset AD [38, 43].

In our group of patients, there is no confirmatory evidence that dural grafts, a possible source of Aβ seeds, were used during neurosurgery. Thus, whilst in some patients with iCJD Aβ transmission has occurred through insertion of contaminated dural grafts [7, 18, 20, 28], alternative routes of Aβ transmission need to be considered.

As described above, patients from our cohort had undergone neurosurgical interventions during childhood for various reasons, and only one had a history of head trauma. The history of a single episode of head trauma in all previously reported young-onset CAA male patients [15, 36, 39] has raised speculations as to the causal relation between male gender, traumatic brain injury (TBI) and development of Aβ pathology. Head trauma, however, is a common cause of morbidity and mortality in children worldwide and a proportion of them require neurosurgical intervention for either skull fracture or associated brain injury [5]. Furthermore, male preponderance in patients with head trauma is well documented in the literature, and the male–female ratio increases with age [47]. Nevertheless, it has long been debated if a history of head trauma, either single and severe, or mild and repetitive, could represent a risk factor for AD, often with contradictory findings between studies, due to different inclusion criteria and outcome measures. In patients with dementia and a history of chronic mild repetitive head trauma, the neuropathological changes have been characterised in detail [32]: the hallmark pathology in these patients is the accumulation of hyper-phosphorylated tau. As these neuropathological changes are distinct from those in AD and, in fact, from any other primary tauopathy, this condition has been termed chronic traumatic encephalopathy (CTE). Recently CTE with concomitant pathologies, such as TDP43 pathology, primary tauopathy, Lewy body pathology and AD have been reported in demented retired soccer players [30]. Expectedly, the majority of elderly patients with at least intermediate level of AD neuropathological change and a history of chronic mild repetitive head injury [30] also had concomitant widespread CAA, as also seen in patients with sporadic AD and no history of head trauma. In a study by Stein et al. [44], Aβ pathology, reported in approximately half of patients with CTE, was strongly associated with the presence of an *APOE* ε4 allele and older age at symptom onset. Whilst these data [44] indeed support that Aβ pathology occurs more frequently in elderly individuals with a history of chronic mild repetitive head trauma, when compared with the normal ageing population, there is no robust evidence for a similar association in patients with history of a single episode of traumatic brain injury. Whilst parenchymal Aβ can be found at early stages after brain trauma [41], only a trend towards increased Aβ plaque pathology has been reported as a long-term consequence after a single TBI in a proportion of patients [24]. Specifically, CAA has been reported only in few patients with fatal head injury, predominantly in individuals carrying at least one *APOE* ε4 allele [25, 29], suggesting that these patients probably had CAA prior to the trauma and that the actual traumatic insult causing brain contusions and haemorrhages leading to a poor outcome was aggravated by the pre-existing *APOE* ε4-driven CAA. Importantly, no association between self-reported history of TBI and risk of development of autopsy-confirmed amyloid angiopathy was found in a large cohort of 213 individuals (*P* > 0.09, Table 4 in [12]). Similarly, no increase of amyloid levels was found in 74 cognitively normal individuals with self-reported history of TBI using PiB-PET imaging [33].

A recent post-mortem study of patients suffering from schizophrenia who underwent leucotomy, which can be considered as a form of single traumatic brain injury with a severe axonal damage, has demonstrated scattered Aβ plaques restricted to the lesional site only in patients with the *APOE* ε4 genotype and minimal CAA in 2 of 10 patients with *APOE* ε3/ε4 genotype (67 and 86 years old with and without leucotomy, respectively) [42]. These findings argue against the notion that mechanical damage through a neurosurgical procedure could have interfered with glymphatic or perivascular Aβ clearance, making neurosurgery per se an unlikely risk factor for the development of widespread CAA later in life. Likewise, a much higher prevalence of CAA would be expected if Aβ oligomerisation and propagation was induced by diathermy or by various haemostatic sealants.

Remarkably little tau pathology in our patients and in published iCJD cohorts [7, 20, 22, 28, 40], suggests, amongst other possibilities, differences in the mechanisms or dynamics of tau transmission.

In conclusion, the history of neurosurgical procedures, the absence of known pathogenic mutations and development of CAA-related brain haemorrhages three decades later raises the possibility that Aβ proteopathic seeds may have been historically transmitted by surgical instruments carrying traces of misfolded Aβ protein. This possibility is underpinned by experimental evidence and case data: intracerebral inoculation of Aβ-rich extracts or implantation of Aβ-coated steel wires into animals caused Aβ pathology [1, 16] and transmission has occurred in humans through administration of cadaveric human growth hormone [7, 22, 40] or dura mater grafting [18, 20, 28]. The increasing frequency of neurosurgical interventions on aged, cognitively intact individuals with cerebral Aβ may pose a risk of onward Aβ proteopathic seed transmission.

Our study is limited by its small size and because it was retrospective could be affected by bias in selection of cases, controls or evidence from the literature. We propose a hypothesis that might be tested by larger epidemiological studies to consider the risk of neurodegenerative disorders after neurosurgery. As it takes more than two decades to develop pathologically detectable Aβ deposits and for CAA to manifest clinically, this has to be considered when planning such epidemiological studies, and it is unlikely that this work can be done prospectively. Any hypothetical transmission during neurosurgery would have occurred at a time when instrument cleaning and sterilisation was done differently from how it is today. Larger, more definitive epidemiological studies are now required to confirm the prevalence of vascular Aβ pathology in patients with a past medical history of neurosurgery. The possibility of transmission of Aβ proteopathic seeds should be taken into account when assessing the safety of surgical procedures, including the adequate sterilisation of reusable surgical instruments [14, 46] and use of disposable instruments where appropriate.

## Electronic supplementary material

Below is the link to the electronic supplementary material.

Supplementary material 1 (DOCX 30 kb)

Supplementary material 2 (XLSX 13 kb)
