# Supplementary material for: Evidence of amyloid-β cerebral amyloid angiopathy transmission through neurosurgery
Source: Acta Neuropathol. 2018 Feb 15;135(5):671–9. doi: 10.1007/s00401-018-1822-2 (PMC5904220; doi:10.1007/s00401-018-1822-2)
Supplement: Supplementary file 1 — Supplementary material 1 (DOCX 30 kb) [file 401_2018_1822_MOESM1_ESM.docx]

**Supplementary table 1. Pathologically confirmed CAA in young adults**

| **Case ID** | **Reference** | **Age** | **Sex** | **Country of presentation** | **Presentation** | **Genetics:**  ***APOEε*** | **Genetics:**  ***APP*,**  ***PSEN1*, *PSEN2*** | **Evidence of neurosurgery in past medical history**  **Estimated interval (Y) from first surgery to diagnosis of CAA** | | **Pathology** | | | | |
| --- | --- | --- | --- | --- | --- | --- | --- | --- | --- | --- | --- | --- | --- | --- |
|  |  |  |  |  |  |  |  |  |  | **CAA** | **Aβ**  **CAA** | **DD** | **MP** | **NP** |
| #1 | Case 1 | 39 | F | Portugal | ICH | 3/4 | No mutations | TBI in childhood requiring multiple neurosurgeries | 36 | + | + | + | +/- | - |
| #2 | Case 2 | 31 | M | Belgium | ICH | 2/3 | No mutations | Neurosurgical resection of intracranial tumour in infancy | 30 | + | + | - | - | - |
| #3 | Case 3 | 36 | F | United Kingdom | ICH | 3/4 | No mutations | Intraventricular shunts, foramen magnum decompression in infancy and childhood | 35 | + | + | + | + | - |
| #4 | Case 4 | 57 | F | United Kingdom | ICH | 3/3 | No mutations | Correction of syringomyelia | 36 | + | + | + | + | +/- |
| #5 | Ehling [15] | 38 | M | Austria | ICH | 3/3 | No mutations | TBI in childhood with osteoclastic craniotomy | >20 | + | + | - | - | - |
| #6 | Purrucker [39] (Case A) | 37 | M | Germany | ICH | NA | *APP* negative;  *PSEN1* and *PSEN2*: NA | TBI in childhood; not documented if surgery performed, but provided imaging suggestive of previous surgery | >20 | + | + | NA | NA | NA |
| #7 | Purrucker [39] (Case B) | 42 | M | Germany | ICH | NA | NA | Penetrating head injury in childhood;  not documented if surgery performed, but provided imaging suggestive of previous surgery | 40 | + | + | NA | NA | NA |
| #8 | Nakayama [36] | 32 | M | Japan | ICH | 3/3 | *NA* | Left frontal subdural haematoma at age of 1; craniotomy and evacuation of haematoma | 31 | + | NA | NA | NA | NA |
| #9 | Wong [49] | 49 | M | China | ICH | NA | NA | No history of head trauma or surgery | NA | + | NA | NA | NA | NA |
| #10 | Campbell [8] | 53 | M | Italy | ICH | NA | NA | Information not provided | NA | + | + | - | - | - |

**Abbrevations:** ICH = intracerebral haemorrhage; NA = not applicable; CAA = cerebral amyloid angiopathy; Aβ CAA = cerebral amyloid-β angiopathy; DD = diffuse parenchymal deposits; MP = plaques with central amyloid cores, but absent peripheral rim of hyperphosphorylated tau positive neurites (mature plaques); NP = plaques with central amyloid cores with peripheral concentric rim of hyperphosphorylated tau positive neurites (neuritic plaques); “+” = present; “-“ = absent “+/-“ = present, but scarce

**Supplementary table 2:**

**Details of 50 control patients.**

Column A, ID of 50 consecutively selected patients as specified in Methods. Columns B, C, gender and age at surgery. Column D, location of the surgical site. Column E, clinical diagnosis. Column F, recent clinical presentation leading to neurosurgery. Column G, past medical history (PMH). Column H, information about previous neurosurgery: three patients with confirmed intervention and one patient with no known history of neurosurgery, but evidence of severe head trauma during childhood was identified (highlighted); in all other patients neurosurgical intervention was either excluded or there was no indication that these patients may have had neurosurgery. Column I, pathologies are grouped as vascular malformation, reactive changes or developmental cerebral malformation. Column J, type of CNS tissue present in the sample. Columns K, L, assessment of amyloid-β in brain parenchyma and blood vessels. Column M, specimen size: small - up to approximately 5x5mm; medium - up to approximately 15x15mm; large - more than 15x15mm. Column N, estimated proportion of CNS tissue in the sample (%).
